# An Investigation of Descending Pain Modulation in Women With Provoked Vestibulodynia: Alterations of Brain Connectivity

**DOI:** 10.3389/fpain.2021.682484

**Published:** 2021-06-17

**Authors:** Lindsey R. Yessick, Caroline F. Pukall, Gabriela Ioachim, Susan M. Chamberlain, Patrick W. Stroman

**Affiliations:** ^1^Department of Psychology, Queen's University, Kingston, ON, Canada; ^2^Centre for Neuroscience Studies, Queen's University, Kingston, ON, Canada; ^3^Department of Obstetrics and Gynecology, Queen's University, Kingston, ON, Canada

**Keywords:** Provoked Vestibulodynia, brain, pain, chronic pain, neuroimaging

## Abstract

Provoked Vestibulodynia (PVD) is the most common vulvodynia subtype (idiopathic chronic vulvar pain). Functional magnetic resonance imaging (fMRI) studies indicate that women with PVD exhibit altered function in a number of pain modulatory regions in response to noxious stimulation, such as in the secondary somatosensory cortex, insula, dorsal midcingulate, posterior cingulate, and thalamus. However, previous neuroimaging studies of PVD have not examined periods of time before and after noxious stimulation or investigated functional connectivity among pain modulatory regions. Fourteen women with PVD and 14 matched Control participants underwent five fMRI runs with no painful stimuli interleaved randomly with five runs with calibrated, moderately painful heat stimuli applied to the thenar eminence. As recent findings indicate that pain processing begins before and continues after painful stimulation, 2-min periods were included in each run before and after the stimulus. Functional brain connectivity was assessed during both trials of Pain and No Pain stimulation for each group using structural equation modeling (SEM). Analyses of variance (ANOVAs) on connectivity values demonstrated significant main effects of study condition, and group, for connectivity among pain modulatory regions. Most of the differences between the Pain and No Pain conditions found only in the PVD group take place before (i.e., thalamus to INS, ACC to S1, thalamus to S1, and thalamus to S2) and after pain stimulation (i.e., INS to amygdala, PPC to S1, and thalamus to S2). Such differences were not observed in the Control group. These findings further support previous results indicating that women with PVD have altered pain processing compared to pain-free women.

## Introduction

Provoked Vestibulodynia (PVD) is the most common subtype of vulvodynia, defined as idiopathic chronic vulvar pain (1). PVD is characterized by provoked pain in the vulvar region, specifically around the vaginal opening (i.e., the vulvar vestibule). Women with vulvodynia exhibit vulvar allodynia and generally have lower sensory thresholds in the vulva than women without PVD (2–4). This hypersensitivity is also present in many non-vulvar regions (e.g., thumb, forearm, deltoid, shin) (2–6). This line of research suggests that factors involving the central nervous system (i.e., brain and spinal cord) and descending influence from the brain on spinal cord excitability (i.e., descending modulation of pain) may play a role in the expression of PVD, a hypothesis that has been further strengthened by neuroimaging studies.

A number of studies to date have employed magnetic resonance imaging (MRI) to investigate PVD (7–13), and the first of these found that women with PVD exhibited increased neural activity in brain regions previously found to play a role in pain processing and the top-down modulation of pain (11). Most recently, a study of women with Genito-Pelvic Pain/Penetration Disorder (GPPPD), a diagnosis that includes PVD, reported that brain responses during a painful stimulus applied to the vestibule were greater in regions primarily related to cognitive and affective functioning known to continuously modulate pain, in comparison to non-affected women. However, the results did not indicate any between-group differences during a period of pain anticipation (10). In addition, neural activity in response to painful stimulation of non-vulvar areas has also been examined. Brain responses of women with vulvodynia, some of whom were diagnosed with PVD, were examined in response to a painful stimulus applied to the thumb (9). Women with vulvodynia were found to have greater ipsilateral insular response, in addition to greater response to “slightly intense” and “painful” thumb pressure in regions related to the experience of pain and the sensory integration of pain (14, 15) as compared to control women (9). It has also been shown that in women with PVD, during task-free functional magnetic resonance imaging (fMRI), there are alterations in neural networks related to sensorimotor processing, the default mode, and salience (7). These networks have been found to have similar, as well as distinct, alterations in patients with other chronic pain disorders (16–18), some of which are common comorbidities of PVD (e.g., fibromyalgia) (19, 20). Taken together, these results suggest that women with PVD may differ from non-affected women in their central modulation of pain and sensory stimuli.

The goal of the present study was to further examine how women with and without PVD differ in central processing of painful stimuli, specifically descending modulation, using a comprehensive connectivity network model of relevant pain processing regions. We hypothesized that women with PVD will exhibit altered descending modulatory processes within an *a priori* model of brain regions known to be related to pain processing. Specifically, we hypothesize that functional connectivity changes for women with PVD in comparison to pain-free Control women will be continuously present before, during, and after pain stimulation.

## Methods

### Participants

Participants were recruited through the Sexual Health Research Laboratory (SHRL) participant database, Kingston community advertisements, social media (e.g., Twitter), pamphlets placed in clinics, and health care providers (e.g., urologists, gynecologists, pelvic health physical therapists). This study was approved by the Queen's University Health Sciences Research Ethics Board and informed consent was obtained.

**Figure 1:**
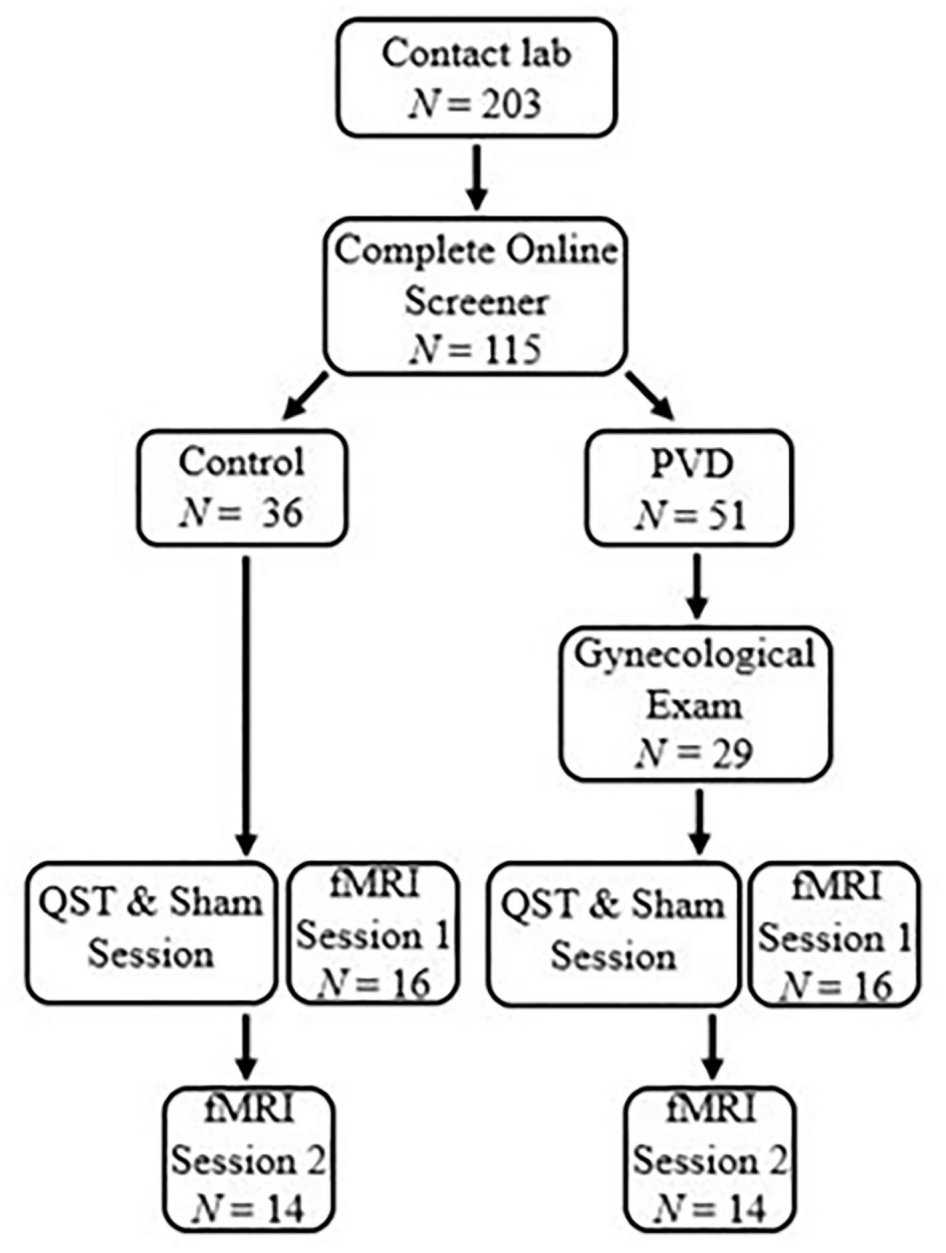
Participant flow chart. Participants completed a survey to determine eligible participants and grouping by contacting the lab. A gynecological exam was performed for women with PVD to confirm PVD symptoms. The first session for both groups began with a QST and Sham session to titrate paradigm heat stimulation and train participants in the paradigm. After the QST and Sham session, participants were immediately randomized into either a brain or BS/SC fMRI session. Participants would then return on a second occasion to complete the second fMRI session. The scope of this paper includes the results of the brain session.

Participant eligibility was assessed either over the phone or online using Qualtrics survey software. Demographic information for the 28 women, 14 in each group (PVD, Control), can be found in Table 1. *T*-tests were used for continuous variables and Fisher's exact tests were used for categorical variables to examine any demographic differences between women with PVD and Control women (Table 1). Groups were matched on age (+/– 5 years) to account for age-related changes in sensory processing and on hormonal contraceptive use (yes or no) to account for any potential effects (e.g., on pain sensitivity) of exogenous hormones (21–23). In order to be eligible, participants were required to be between the ages of 18 and 50; not currently pregnant, breastfeeding, or using medications that substantially affect the central nervous system (e.g., antipsychotics); and have no magnetic resonance imaging (MRI) contraindications (e.g., metal implants) or major brain or spinal cord injury. Women with PVD were also required to report idiopathic, provoked pain at the vaginal entrance and a non-zero average pain intensity rating during a cotton-swab palpation of the vestibule (see below). Participant responses to the screening questions and the results of the gynecological examination (see below) formed the basis to determine inclusion for women with PVD. The primary exclusion criterion for Control women was a history of, or current, chronic vulvar pain. As this study was part of a larger study collecting data on spinal cord imaging that involved heat stimulation to the right hand, we chose the same location. For this reason, the right hand was used for stimulation in all participants and handedness was not screened for eligibility in this study design. Figure 1 illustrates the number of participants who completed each phase of participation.

**Table 1:** Participant demographic information.

	**PVD sample *n* = 14**	**Control sample *n =* 14**	**Total sample *n* = 28**	***p*-value**
Age [*M* (*SD*)]	31.0 (10.0)	30.6 (10.2)	30.8 (9.9)	0.835
Sexual orientation [*n* (%)]				0.098
Heterosexual	14 (100.0)	10 (71.4)	24 (85.7)	
Bisexual		2 (14.3)	2 (7.1)	
Same sex attracted		1 (7.1)	1 (3.6)	
Not sure		1 (7.1)	1 (3.6)	
Relationship status [*n* (%)]				0.836
Married	6 (42.9)	2 (14.3)	8 (28.6)	
Dating partner (regularly)	2 (14.3)	2 (14.3)	4 (14.3)	
Common-law	2 (14.3)	2 (14.3)	4 (14.3)	
Single (not dating)	1 (7.1)	2 (14.3)	3 (10.7)	
Casual sex (one partner)	1 (7.1)	1 (7.1)	2 (7.1)	
Dating partner (long distance)	1 (7.1)	2 (14.3)	3 (10.7)	
Living with partner	1 (7.1)	2 (14.3)	3 (10.7)	
Casual sex (multiple partners)		1 (7.1)	1 (3.6)	
Birthplace [*n* (%)]				0.472
Canada	11 (78.6)	11 (78.6)	22 (78.6)	
Latin/South America	2 (14.3)		2 (7.1)	
United States	1 (7.1)	1 (7.1)	2 (7.1)	
Europe		2 (14.3)	2 (7.1)	
Ethnicity [*n* (%)]				0.013
Canadian	11 (78.6)	7 (50.0)	18 (64.3)	
Latin	2 (14.3)		2 (7.1)	
North American Indigenous	1 (7.1)		1 (3.6)	
European		3 (21.4)	3 (10.7)	
Canadian Arabic		1 (7.1)	1 (3.6)	
Asian		3 (21.4)	3 (10.7)	
Education [*n* (%)]				0.652
High school (complete)	1 (7.1)		1 (3.6)	
College/undergraduate degree (some)	2 (14.3)	5 (35.7)	7 (25.0)	
College/undergraduate degree (complete)	6 (42.9)	4 (28.6)	10 (35.7)	
Graduate/professional (some)	2 (14.3)	1 (7.1)	3 (10.7)	
Graduate/professional (complete)	3 (21.4)	4 (28.6)	7 (25.0)	
Income [*n* (%)]				1.00
$0–9,999	2 (14.3)	1 (7.1)	3 (10.7)	
$10,000–19,999	2 (14.3)	1 (7.1)	3 (10.7)	
$20,000–29,999	1 (7.1)	2 (14.3)	3 (10.7)	
$30,000–39,999	1 (7.1)	1 (7.1)	2 (7.1)	
$40,000–49,999		1 (7.1)	1 (3.6)	
$50,000–59,999	1 (7.1)	2 (14.3)	3 (10.7)	
$60,000 +	5 (35.7)	5 (35.7)	10 (35.7)	
History or current chronic pain [*n* (%)]				
Back/neck/shoulder pain	2 (14.13)			
Migraines	3 (21.3)			
Pain related to ovarian cysts	1 (7.1)			
Radiculopathy	1 (7.1)			

*Due to missing data, multiple responses, and rounding, not all percentages add up to 100*.

### Gynecological Exam

Because vulvodynia is a diagnosis of exclusion (1), the research gynecologist ruled out factors known to be related to vulvar pain (e.g., infections) for participants believed to have PVD. The gynecologist visually and manually examined the internal and external genitals and reproductive organs. Participants were asked to self-report their history of sexually transmitted (e.g., chlamydia, gonorrhea, herpes, HPV) and other (e.g., yeast) infections, and the study gynecologist indicated any sign of infection based on a visual and manual inspection. The cotton-swab test of the external genitals—the main gynecological test for the diagnosis of PVD—was conducted to confirm pain localization (24). The gynecologist palpated the labia majora, inner labia minora, midline areas, and six randomly ordered locations at the vestibule (e.g., 1, 4, 6 o'clock). After each palpation, participants rated their pain intensity on a scale from 0 (no pain at all) to 10 (worst pain ever felt). To be eligible for the study, those with PVD were required to report a mean average pain intensity rating of at least 1/10 during the cotton-swab test of the vestibule and were only required to report pain in at least one location. However, all participants in this study reported pain in four or more locations.

### Quantitative Sensory Testing and Sham MRI

The Queen's MRI Facility Sham Room was used for the QST training and sham MRI session. This room was designed to be similar to the actual MRI environment to acclimate participants to the MRI environment without the magnetic field. The purpose of this room, in which the QST training and “runs” in the mock scanner took place, was to familiarize participants with the MRI environment and protocol in order to reduce anxiety so that more consistent functional magnetic resonance imaging (fMRI) results can be obtained across repeated runs. Four participants in the PVD group completed the QST training but were not able to take part in the mock scanner practice runs as a result of time constraints. Analyses of data with and without these participants revealed no significant differences, and all participants were included in analyses.

The QST training sessions proceeded as follows. First, for each participant, a “moderately painful” temperature was determined on a 101-point scale with verbal descriptors in increments of 10 (i.e., 0 = no sensation, 10 = warm, 20 = a barely painful sensation, 30 = very weak pain, 40 = weak pain, 50 = moderate pain, 60 = slightly strong pain, 70 = strong pain, 80 = very strong pain, 90 = nearly intolerable pain, 100 = intolerable pain). In the instance that participants rated up to the safety limit of 51°C as less than “moderately painful,” then 51°C was used during trials. Noxious heat stimulation was delivered to the participant's right hand using an MRI-compatible robotic contact-heat stimulator (RTS-1; Spinal Map Inc., Kingston). The robotic thermode is within a plexi-glass case and rises through a cut-out to contact the heel of the right thumb (corresponding to the C6 dermatome) and is then lowered again, with the timing, duration of contact, and temperature controlled by custom-made software. Initially, participants received three contacts lasting for 1.5 s at 45°C and were asked to rate each individual sensation. After 2 min of rest to avoid sensitization, this procedure was repeated with 46 and 47°C. This task was intended to train participants to rate the sensory experience. Participants then rated the sensation intensity for 10 consecutive thermode contacts, with onsets every 3 s, at different temperatures (i.e., 46 C, 50 C, 44 C, and 48 C). Each set of 10 contacts was followed by a 2-min rest period to avoid sensitization. Repeated applications of brief stimuli can evoke temporal summation of second pain, which is mediated by C-fibers, and provides a robust BOLD response for fMRI studies of pain (25–28). This procedure enabled us to determine each participant's pain sensitivity and the appropriate temperature for stimulation during the fMRI session.

### MRI Acquisition Protocols

A research-dedicated 3 tesla Siemens Magnetom Trio was used for imaging. To reduce excessive movement and increase comfort, participants were positioned on their back with padding and blankets. The MRI signal was detected with a 12-channel head coil. Participants could view the rear-projection screen, on which the pain rating scale and instructions were displayed using a mirror positioned over their head. The protocol consisted of one structural and 10 4.5-min functional scans (i.e., “runs”), with 2-min breaks between runs. For subsequent slice positioning, initial localizer images were acquired in three planes spanning the entire brain. Functional MRI (fMRI) data for the brain were acquired with gradient-echo echo-planar imaging (EPI), with a 64 × 64 matrix and 192 × 192 mm field-of-view, with 3 mm thick contiguous slices yielding 3 mm cubic voxels, totaling 49 slices spanning the brain. The echo time (TE) was 30 ms and the repetition time (TR) was 3 s with a flip angle of 90°. Ninety volumes were collected for each run.

### fMRI Experimental Design

**Figure 2:**
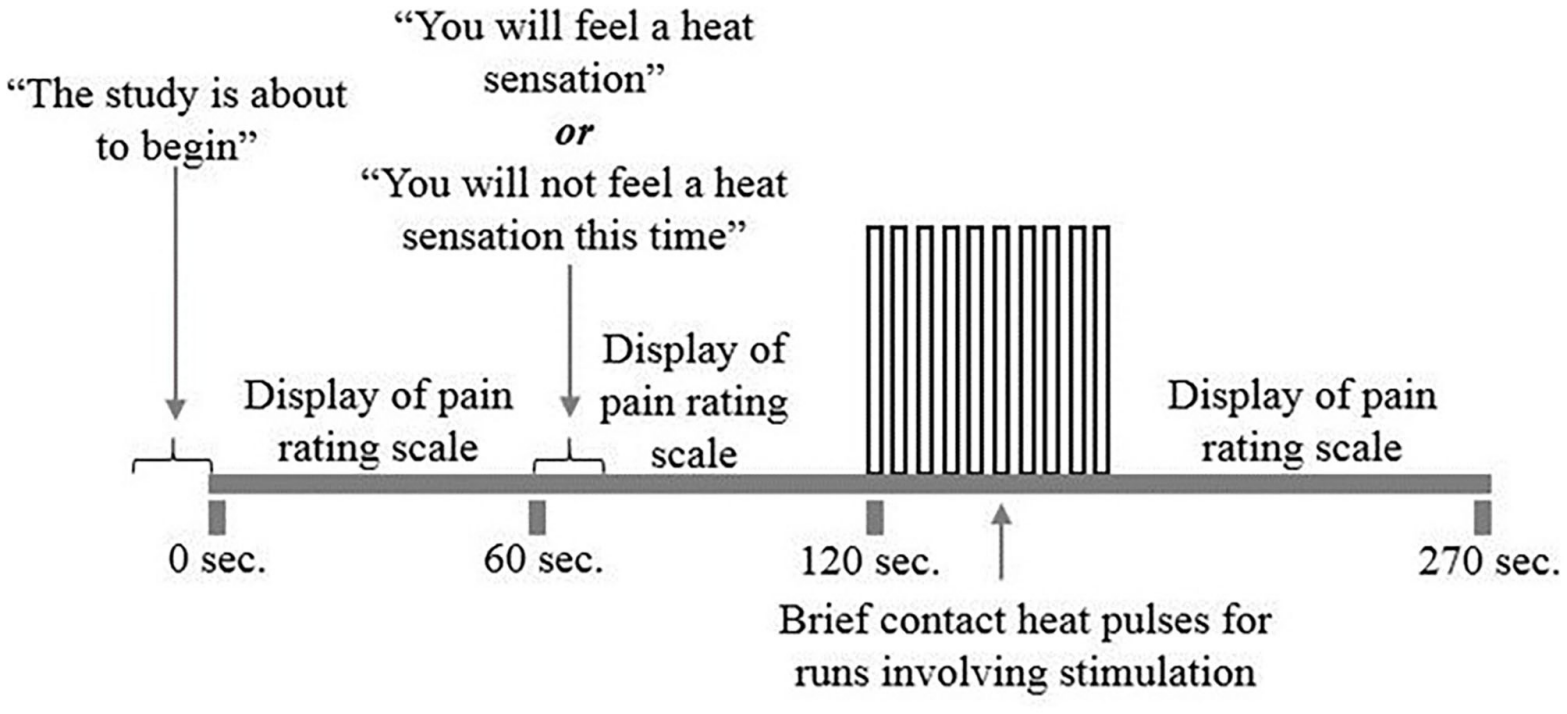
fMRI study design. The study included 10 runs of the study paradigm, five runs with and without pain stimulation. Participants would first be told that the run was about to begin. A pain rating scale was displayed on the screen for participants to reference. One minute into the run, participants were told whether or not that run would include heat stimulation. Two minutes into the run, participants received a total of 10 repeated heat contacts titrated to a moderately painful temperature for 30 s. If the run did not include heat stimulation, participants were at rest during this 30 s period. A 2-min rest would follow each heat stimulation period while the run and scanning completes.

The protocol consisted of a total of 10 runs, 5 runs with pain stimulation and 5 runs without pain stimulation, pseudo-randomized to interleave trial types. For each run, participants were told that a new run was about to begin but were not initially informed if the run would be a Pain or No Pain condition. The study paradigm (Figure 2) consisted of an initial period without stimulation. One minute after the start of the run, participants were told if they would be receiving heat stimulation or not. If the run consisted of pain stimulation, beginning 2 min after the start of the run, the participant would receive 10 heat contacts that elicited a predetermined moderate level of pain to the heel of the right thumb, over a 30-s period. This was followed by a 2-min period of data acquisition following pain stimulation in which participants rested. Each run took a total of 4.5 min to complete. Participants silently rated their pain for each contact and were asked to report their ratings for the first and last contact at the end of each run. If the run consisted of the No Pain condition, the participants were similarly informed 1 min after the start of the run, and otherwise rested during the 4.5-min data acquisition. There were 2-min breaks between runs to avoid skin sensitization.

### Study Procedure

Participants with PVD who were initially eligible based on the screening were scheduled for a gynecological examination to confirm their eligibility. Women with PVD gave informed consent at the gynecological exam and again at the MRI session, and Control women gave informed consent at the MRI session, prior to the QST training and Sham MRI session. They were asked to refrain from alcohol for at least 12 h prior to the imaging portion of the study and from caffeine for at least 6 h before the study, as these substances may affect mood, alertness, and the BOLD response. Participants were also asked to eat a regular meal at their usual mealtime time prior to participation in the study.

Prior to the imaging session (1.5 h), the QST/sham MRI session was conducted (1 h). Before entering the magnetic imaging environment, participants again confirmed that they had no contraindications for the subsequent magnetic resonance imaging. Participants were asked to change into MRI-safe clothing provided by the facility if any item of clothing possibly contained materials that were not MRI safe. They were then positioned in the MRI for scanning to commence. At the end, participants were debriefed and compensated. This study was part of a larger study that involved a separate session of brainstem and spinal cord imaging (29), saliva collection, and the administration of validated self-report measures; these additional components are not discussed in this paper.

### Data Processing

Statistical Parametric Mapping (SPM12) software was used to preprocess the fMRI data (Wellcome Trust Center for Neuroimaging, Department of Imaging Neuroscience, London, UK; http://www.fil.ion.ucl.ac.uk/spm). Imaging data were first converted to Neuroimaging Informatics Technology Initiative (NIFTI) format. Data were then realigned (co-registered) for motion correction, slice-timing correction was applied to correct for differences in image acquisition time between slices and were then spatially normalized to the Montreal Neurological Institute (MNI) standard space. Co-registration parameters were used to model bulk motion to account for residual motion effects, by fitting and subtracting these terms from the image data.

### Structural Equation Modeling

**Figure 3:**
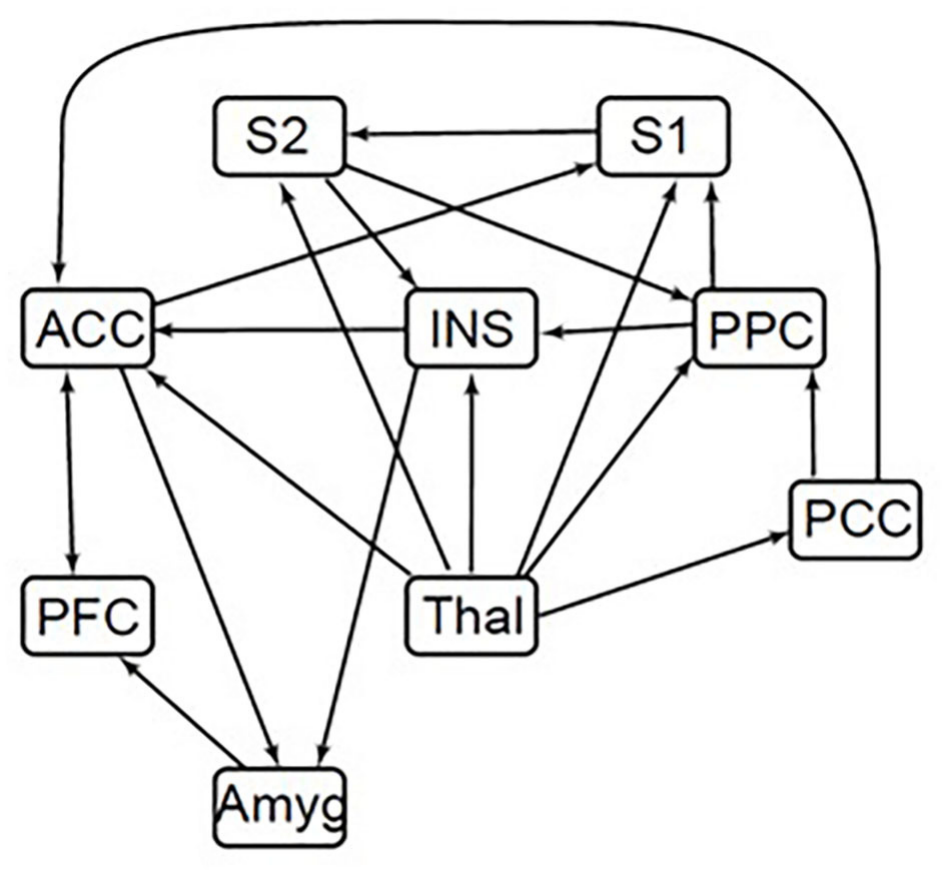
Brain SEM model. ACC, anterior cingulate cortex; INS, insular cortex; PCC, posterior cingulate cortex; PFC, prefrontal cortex; Thal, thalamus; Amyg, amygdala; PPC, posterior parietal cortex; S1, primary somatosensory cortex; S2, secondary somatosensory cortex. Directionality for each connection is indicated by arrows in the defined model. A connection with only a single arrow in the model indicates only one direction modeled. Arrows moving both toward and away from a region indicates either direction.

SEM was used to assess functional connectivity with custom-made software written in MATLAB (30). The adapted SEM procedure required a predefined model that represents plausible interactions of brain regions related to pain processing to constrain the number of possible results (30). The a priori model of anatomical connections and regions known to be related to pain processing is based on known neuroanatomy (15), and includes the amygdala, thalamus, posterior parietal cortex (PPC), prefrontal cortex (PFC), anterior cingulate cortex (ACC), insular cortex (INS), posterior cingulate cortex (PCC), and the primary (S1) and secondary somatosensory cortices (S2) (Figure 3). The anatomical model provides additional information, enabling the inference of directionality for interactions, including both ascending and descending modulation. In order to reduce the number of comparisons that would be necessary for voxel-to-voxel comparisons, these regions of interest were divided into seven subdivisions using k-means clustering based on voxel time-series data. With this clustering method, time-series properties of the MRI signal were used to group voxels with similar blood-oxygenation-level dependent (BOLD) responses, and to separate them from voxels containing primarily noise and increase anatomical precision. The subdivisions were thus based on functional characteristics, not known anatomical subdivisions, and the same subdivisions were used for all groups and conditions.

The anatomical model used for SEM identifies the source regions which may provide input to each target region. SEM was carried out by means of a general linear model to calculate the connectivity strength (i.e., linear weighting factors, termed β-values) of each source region's input to a specified target region. Connectivity values (β) were computed for each participant, using data from all runs of the same type. However, β-values were allowed to vary dynamically between different periods of the stimulation paradigms, by calculating the values using data within selected time periods (epochs) spanning 45 s. SEM analyses were applied to three selected time periods: before the stimulation began (75–120 s), spanning the stimulation period (115–160 s), and after stimulation had ended (153–198 s). Values were also computed for every possible combination of subdivisions within each region, in order to identify the subdivisions which provided the best fit, with the chosen anatomical model. The goodness-of-fit was calculated by examining the proportion of the variance in the target region that was accounted for (i.e., the *R*^2^ value). The significance of the fit was determined by converting R-values to Z-scores (using Fisher's transform; Z = tanh^−1^ (R)$\sqrt{tsize}-3$). *Z*-value distributions have been computed previously, for different network parameters, to identify the probability of a given Z-score occurring by random chance (30), and significance was inferred at a family-wise error-rate corrected *p*_fwe_ < 0.05. The regions/subdivisions that best fit the anatomical model were thus identified for each run type, both for women with PVD and healthy Control women. Using a *t*-test, we determined the significance of the group average β-values based on their estimated standard error across participants, compared to the null hypothesis (i.e., β = 0).

An analysis of variance (ANOVA) was used to examine SEM weighting factor variations between the study groups (PVD or Control) and conditions (Pain or No Pain). First, connections with significant fits (*p*_fwe_ < 0.05) in any of the four combinations of study group and conditions were selected. A 2 × 2 ANOVA consisting of study conditions and groups as the independent variables was then used to analyze the β-values from all participants for each connection. The significant main effect of each independent variable and their interaction was concluded at a family-wise-error corrected *p* < 0.05.

The SEM and ANOVA analyses reported in the present study examine brain connectivity using a data-driven approach to provide greater information to the BOLD responses observed in brain regions relevant to pain processing (31, 32). For this reason, the study results are summarized by connectivity strengths between regions and not by relative BOLD responses within each region of interest.

## Results

### Demographic Characteristics

The groups significantly differed in their self-identified ethnicity, with the majority of participants with PVD identifying as Canadian (11/14) compared to the Control participants, who identified primarily as Canadian (7/14), European (3/16), Canadian Arabic (1/16), and Asian (3/16) (Table 1). Due to practical restraints of the scheduling organization within the MRI facility we were unable to control for phase of menstrual cycle. However, a Fisher's exact test of self-report menstrual cycle phase (i.e., follicular, ovulatory, luteal, menstrual, or no longer menstruating) revealed no significant differences between women with PVD and Control women, *p* = 0.969.

### QST Results

Table 2 summarizes the pain ratings and temperatures of the stimulus used during the imaging sessions as group averages across individual participants. Although participants with PVD reported moderate levels of pain at lower temperatures than Control participants (i.e., women with PVD exhibited lower pain thresholds than Control women), this difference was not statistically significant when compared with a *t*-test.

**Table 2:** Average temperatures and pain ratings during MRI sessions.

	**PVD sample *n* = 14**	**Control sample *n =* 14**	***p*-value**
Average temperature	48.7 (2.1)	49.5 (1.1)	0.225
Average pain ratings	46.1 (10.7)	47.4 (9.4)	0.661

### Group Level SEM Results

The SEM results showed extensive network connectivity between brain regions in all three time periods. Network connectivity was compared between the four participant groups: PVD women experiencing pain (PVD Pain) or no stimulus (PVD No Pain), and Control women experiencing pain (Control Pain) or no stimulus (Control No Pain). Table 3 summarizes the significant differences in connectivity strengths for each group and condition, in the three time periods.

**Table 3:** Summary of significant differences in brain connectivity between all groups, analyzed with SEM.

**Time period**	**Region source → target**	**Control pain**	**Control no pain**	**PVD pain**	**PVD no pain**
**Control pain vs. control no pain**					
During stimulation	ACC → amygdala	**−0.06** **±** **0.01**	**−0.01** **±** **0.01**	−0.03 ± 0.02	−0.03 ± 0.02
	Thalamus → S1	**0.10** **±** **0.01**	**0.06** **±** **0.01**	−0.03 ± 0.01	−0.04 ± 0.01
**PVD pain vs. PVD no pain**					
Before stimulation	Thalamus → INS	0.38 ± 0.07	0.40 ± 0.07	**0.08** **±** **0.08**	**0.40** **±** **0.08**
	ACC → S1	0.18 ± 0.03	0.29 ± 0.03	**0.08** **±** **0.03**	**0.27** **±** **0.04**
	Thalamus → S1	0.07 ± 0.01	0.01 ± 0.01	**-0.00** **±** **0.01**	**-0.06** **±** **0.01**
	Thalamus → S2	0.37 ± 0.08	0.43 ± 0.07	**0.82** **±** **0.08**	**0.54** **±** **0.06**
During stimulation	S2 → PPC	0.40 ± 0.07	0.39 ± 0.12	**0.25** **±** **0.05**	**0.55** **±** **0.08**
	PPC → S1	0.54 ± 0.07	0.49 ± 0.04	**0.72** **±** **0.07**	**0.32** **±** **0.06**
	Thalamus → S2	0.51 ± 0.11	0.29 ± 0.09	**0.65** **±** **0.10**	**0.22** **±** **0.08**
After stimulation	INS → amygdala	0.28 ± 0.07	0.22 ± 0.05	**0.37** **±** **0.09**	**0.07** **±** **0.07**
	PPC → S1	0.54 ± 0.07	0.49 ± 0.04	**0.72** **±** **0.07**	**0.32** **±** **0.06**
	Thalamus → S2	0.51 ± 0.11	0.29 ± 0.09	**0.65** **±** **0.10**	**0.22** **±** **0.08**
**Control no pain vs. PVD no pain**					
Before stimulation	INS → amygdala	0.28 ± 0.05	**0.27** **±** **0.06**	0.21 ± 0.08	–**0.08** **±** **0.08**
	Thalamus → S1	0.07 ± 0.01	**0.01** **±** **0.01**	−0.00 ± 0.01	–**0.06** **±** **0.01**
	PPC → S2	0.76 ± 0.05	**0.73** **±** **0.05**	0.25 ± 0.07	**0.23** **±** **0.07**
	S1 → S2	−0.17 ± 0.05	**-0.20** **±** **0.04**	0.05 ± 0.10	**0.25** **±** **0.08**
**Control pain vs. PVD pain**					
Before stimulation	Amygdala → PFC	**0.58** **±** **0.08**	0.32 ± 0.07	**0.24** **±** **0.05**	0.16 ± 0.05
	Thalamus → ACC	–**0.05** **±** **0.01**	−0.03 ± 0.01	**0.01** **±** **0.01**	0.02 ± 0.02
	Thalamus → PCC	**1.07** **±** **0.02**	1.05 ± 0.02	**0.69** **±** **0.03**	0.68 ± 0.02
	S2 → PPC	**1.07** **±** **0.02**	1.12 ± 0.02	**1.18** **±** **0.03**	1.19 ± 0.03
	PPC → S1	**0.55** **±** **0.05**	0.53 ± 0.05	**0.80** **±** **0.04**	0.69 ± 0.05
	Thalamus → S1	**0.07** **±** **0.01**	0.01 ± 0.01	–**0.00** **±** **0.01**	−0.06 ± 0.01
	PPC → S2	**0.76** **±** **0.05**	0.73 ± 0.05	**0.25** **±** **0.07**	0.23 ± 0.07
	Thalamus → S2	**0.37** **±** **0.08**	0.43 ± 0.07	**0.82** **±** **0.08**	0.54 ± 0.06
	INS → amygdala	**0.22** **±** **0.03**	0.28 ± 0.03	**0.36** **±** **0.03**	0.35 ± 0.04
	ACC → amygdala	**0.31** **±** **0.03**	0.26 ± 0.04	**0.15** **±** **0.04**	0.18 ± 0.04
During stimulation	Amygdala → PFC	**0.44** **±** **0.07**	0.31 ± 0.07	**0.07** **±** **0.05**	0.09 ± 0.06
	Thalamus → ACC	–**0.05** **±** **0.01**	−0.05 ± 0.01	**0.03** **±** **0.02**	0.01 ± 0.01
	Thalamus → PCC	**1.03** **±** **0.02**	1.08 ± 0.02	**0.70** **±** **0.03**	0.66 ± 0.03
	S2 → PPC	**0.97** **±** **0.02**	1.11 ± 0.02	**1.13** **±** **0.03**	1.17 ± 0.03
	PPC → S1	**0.41** **±** **0.05**	0.56 ± 0.04	**0.79** **±** **0.04**	0.70 ± 0.04
	Thalamus → S1	**0.10** **±** **0.01**	0.06 ± 0.01	–**0.03** **±** **0.01**	−0.04 ± 0.01
	PPC → S2	**0.76** **±** **0.06**	0.74 ± 0.05	**0.21** **±** **0.07**	0.13 ± 0.08
	Thalamus → S2	0.39 ± 0.11	0.41 ± 0.07	0.97 ± 0.08	0.59 ± 0.07

*Columns outline the weighting factor (β) of the specific connection and the respective error, while bolded pairs in the columns denote which pairs of β-values are significantly different. Only connections with significant differences in at least one comparison of group/condition are shown here. ACC, anterior cingulate cortex; INS, insular cortex; PCC, posterior cingulate cortex; PFC, prefrontal cortex; PPC, posterior parietal cortex; S1, primary somatosensory cortex; S2, secondary somatosensory cortex*.

Comparisons of network connectivity in Control women in the Control Pain and Control No Pain conditions demonstrated significant differences in strengths (β) of connections from the ACC to the amygdala, and from the thalamus to S1, for the time period corresponding with when the stimulus was applied in the Pain conditions. Comparing women with PVD in the Pain and No Pain conditions, we found differences in connectivity from the thalamus to the insula, S1, and S2, and from the ACC to S1, in the period before stimulation. During the stimulation period, women with PVD had significantly different connectivity for connections from the S2 to the PPC, from the PPC to S1, and from the thalamus to S2. After stimulation, we found differences in connectivity from the insula to the amygdala, from the PPC to S1, and from the thalamus to S2.

In the Pain condition, in the period before stimulation, there were significant differences between the Control and PVD women in connections from the amygdala to the PFC, from the insula and ACC to the amygdala, from the thalamus to the ACC, PCC, S1 and S2, from S2 to the PPC, and from the PPC to S1. During stimulation, connectivity differences between these groups were detected in the connections from the amygdala to the PFC, from the thalamus to the ACC, from the PCC to S1 and S2, from S2 to the PPC, and from the PPC to S1 and S2.

### ANOVA Results

**Figure 4:**
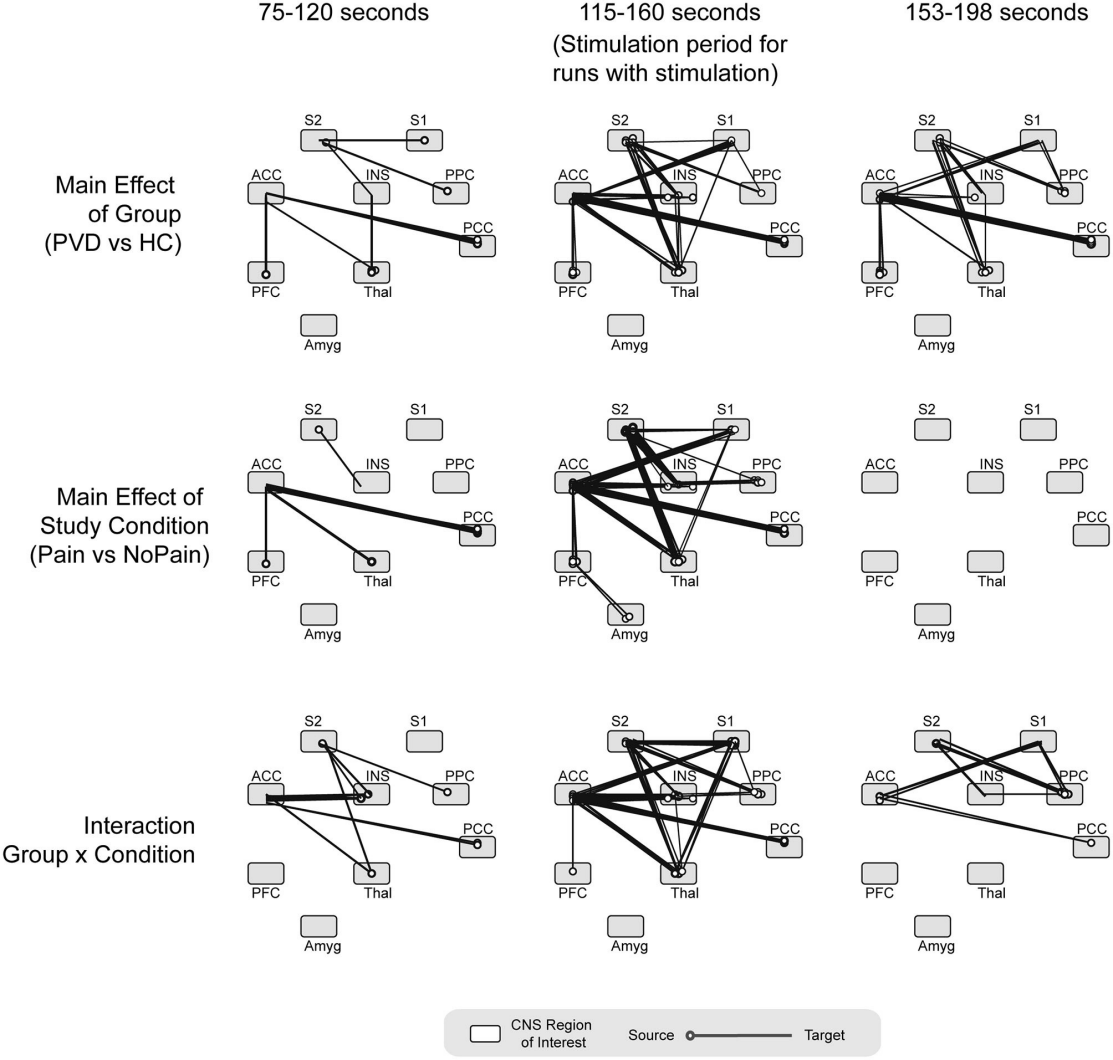
ANOVA results. Summarizing the main effect of group (Control women vs. women with PVD), study condition (Pain vs. No Pain) and group x condition interaction effects, analyzed at three different time points (period before the stimulation, during noxious stimulation, and period after the stimulation). ACC, anterior cingulate cortex; INS, insular cortex; PCC, posterior cingulate cortex; PFC, prefrontal cortex; Thal, thalamus; Amyg, amygdala; PPC, posterior parietal cortex; S1, primary somatosensory cortex; S2, secondary somatosensory cortex. Circles on each region depict the region of origin for each connection. The line extending from the circle indicates the connection's direction.

Results of an analysis of variance (ANOVA) on β-values, with the participant group (Control women vs. women with PVD) and condition (Pain or No Pain) as independent variables, are plotted in Figure 4. These results show several connections for which connectivity strengths varied specifically with the group, as well as connections with connectivity strengths that varied with the stimulation condition. In the period before stimulation, several connections demonstrated a significant group effect (Control compared to PVD), including connections from the PFC and PCC to the thalamus and ACC, from S1 and the PCC to S2, from S2 to the insula, and from the thalamus to the insula. During this period, we also identified connectivity strengths which varied significantly with the condition (Pain or No Pain), that consisted of connections from the thalamus and PFC to the ACC, connections between the ACC and PCC, and connections from S2 to the insula. Interaction effects between the group and study condition were mainly observed in connections from the insula, PCC, and thalamus to the ACC, from the thalamus, insula, and PPC to S2, and from S2 to the insula.

During the stimulation period, we identified several connections for which the connectivity strengths varied significantly with the group, primarily consisting of connections to and from the ACC and S2 regions. Similar connections also had significant condition effects (with connectivity strengths that varied significantly with the study group) as well as interaction effects, with the exception of connections from the amygdala to the PFC which did not have any significant interaction effects. After the stimulation period, similar connections showed a significant group effect as during the stimulation. The primary exception between the stimulation period and the period after stimulation, is that no connections to or from the S1 region had a significant group effect in the period after stimulation. In the period after stimulation, no connections had connectivity that varied with the study condition (condition effects) although several connections showed significant interaction effects, including from the ACC to the PCC and S1, from the PPC to the insula, S1, and S2, and from S2 to the insula.

## Discussion

In this study, an adaptation of structural equation modeling (SEM) examined functional connectivity using a predefined model that represents how regions interact with each other during pain processing. Control participants and women with PVD underwent a functional MRI paradigm to assess functional connectivity during the period of time before, during, and after receiving a pain stimulus, given the importance of pain perception processes when expecting and ruminating about a pain stimulus (33). Both groups also completed trials without a painful stimulus for the comparison of functional connectivity when receiving a pain stimulus vs. activity in the absence of a stimulus. This study was the first to employ a comprehensive network connectivity model to examine coordinated BOLD signal variations amongst regions relevant to pain processing in women with PVD. Moreover, this study is novel because we employed a study design that investigated differences between runs involving moderately painful stimulation and no stimulation.

Based on the SEM connectivity (i.e., β) values presented in Table 3 (Control No Pain vs. PVD No Pain, and Control Pain vs. PVD Pain), our results indicate that women with PVD exhibited significantly different connectivity from Control participants among regions involved in pain processing. During the Pain condition, there were significant differences between Control participants and participants with PVD in a number of connections involving the thalamus, amygdala, PPC, S1, and S2. These regions play a role in the emotion regulation, salience, and sensory integration of pain, and they have been found to have greater connectivity in women with PVD and fibromyalgia (a pain condition commonly comorbid with PVD) (4, 7, 9, 10, 34, 35). In addition, between-group differences in connectivity of the sensorimotor cortex and PPC were found in the present study. Combined with Hampson et al.'s (9) report of a positive association between BOLD responses of the sensorimotor and inferior parietal cortices and the clinical pain experience of women with PVD, this study is further evidence of alterations in pain modulation involving these regions in women with PVD.

Interestingly, the ANOVA results we presented also indicated that a number of connections exhibited significant interactions between groups and conditions, during noxious stimulation. The significant differences between conditions for women with PVD (i.e., S2 to PPC, PPC to S1, thalamus to S2) are different from those for Control participants (i.e., ACC to amygdala, thalamus to S1). The results observed during pain stimulation may provide further support for the conclusion that connectivity of regions associated with pain modulation is altered in women with PVD. In addition to the connectivity differences between and within groups during pain stimulation, most of the differences between the Pain and No Pain conditions that are found only in the PVD group take place *before* (i.e., thalamus to INS, ACC to S1, thalamus to S1, and thalamus to S2) and *after* pain stimulation (i.e., INS to amygdala, PPC to S1, and thalamus to S2). In contrast, Control participants exhibited no such significant differences. Previous research has shown that there is a continuous component to pain regulation, meaning that elements of pain modulation occur before, during, and after receiving noxious pain stimulation—not solely during the experience of pain (33). ANOVA results for the period following pain stimulation revealed no effects on connectivity by condition alone, meaning that connectivity did not differ significantly between the Pain and No Pain conditions. However, connectivity differed significantly between both groups and a significant interaction effect (group x condition) was also identified in several network connections, suggesting that group status had a significant influence over the observed connectivity differences.

The SEM and ANOVA analyses revealed numerous significant between-group differences during all three time periods of the trials (before, during, and after pain stimulation). This pattern is of particular interest considering that women with PVD received comparable temperature levels as Control participants to experience a moderate level of pain. Contrary to expectations, women with PVD reported similar levels of pain during painful stimulation to Control participants. This may indicate that central pain modulation systems are altered in women with PVD, possibly due to differences in the subjective (e.g., affective, motivational) interpretation of the painful stimulus. Indeed, many of the group differences in connectivity featured the ACC as the target region. The ACC is involved in multiple aspects of the subjective experience of pain, specifically the assessment of pain unpleasantness (36).

In addition, models of attention have indicated that the ACC may regulate affective and cognitive processes, suggesting ACC activation in response to pain could reflect the region's role in behavioral and emotional responses and cognitive coping processes (36, 37). Consistent with this link, women with GPPPD, a diagnosis that includes PVD, exhibited greater brain responses in regions primarily related to cognition and affect during painful stimulation in comparison to non-affected women (10). In addition, a study examining functional connectivity in the brainstem and spinal cord in women with and without PVD also revealed significant alterations in regions (e.g., hypothalamus) that share antinociceptive pathways with regions related to affective pain processing (e.g., prefrontal cortex) (29). It may be that women with PVD exhibit alterations in the affective and cognitive components related to coping with and processing painful stimuli in comparison to Control women, as has been demonstrated in the self-report literature (6, 38, 39).

This study advanced our knowledge of PVD by showing new elements of how pain processing and continuous pain modulation are altered in the provoked pain condition. Importantly, these results illustrate that women with PVD differ significantly from healthy women in their central pain processing even though at the time of the study they received the same intensity of the noxious stimulus and were not otherwise in pain.

### Limitations

The heterogeneous nature of PVD (e.g., variance in symptom severity, comorbidities, and psychosocial characteristics) could impact pain perception and processing in women with PVD. In particular, factors related to emotional responses (e.g., catastrophizing) could contribute to alterations in pain processing (40). In addition, a downside of SEM methods is that regions are chosen for analysis of connectivity based on previous descending pain regulation research. Because of this selection method, it is possible that regions related to pain processing were not considered in the connectivity network. However, if we were to include more regions, we risk incorporating non-significant network connections that then impact the strength of each network component.

### Implications and Conclusions

This study is the first to examine a comprehensive connectivity model of pain processing in women with and without PVD. In addition to the effect of study condition, the effect of having PVD, or not, resulted in significant differences in connectivity of regions responsible for pain processing and the cognitive and emotional responses to painful stimulation. Not only did women with PVD exhibit significant differences in connectivity during pain stimulation, but based on the ANOVA results, women with PVD appear to have significant differences in connectivity across pain regions both before and after pain stimulation. This effect appears to be specifically driven by the presence of a PVD diagnosis, as there were fewer significant differences prior to, and no significant differences following, pain stimulation as a result of the condition. This finding is in comparison to group status (PVD or Control), which had a notably greater number of significant differences both before and after pain stimulation. In addition, despite numerous significant between-group differences in connectivity during all three time periods of the trials, women with PVD reported similar levels of pain during painful stimulation to control participants, suggesting that alterations of modulation systems in women with PVD may be due to differences in the subjective interpretation of the painful stimulus. This study adds to the body of research suggesting there are alterations in central pain processing of women with PVD and supplements previous research to show that women with PVD have altered pain processing before, during, and after the experience of pain.

## Data Availability Statement

The raw data will be made available by the authors, without undue reservation is correct.

## Ethics Statement

The studies involving human participants were reviewed and approved by Queen's University Health Sciences and Affiliated Teaching Hospitals Research Ethics Board (HSREB). The patients/participants provided their written informed consent to participate in this study.

## Author Contributions

LY and PS: conceptualization, data acquisition, formal analysis, methodology, writing—original draft, writing—review, and editing. CP: conceptualization, funding acquisition, data acquisition, methodology, resources, supervision, writing—original draft, writing—review, and editing. GI: data acquisition, formal analysis, methodology, writing—review, and editing. SC: conceptualization, data acquisition, writing—review, and editing. All authors contributed to the article and approved the submitted version.

## Conflict of Interest

The authors declare that the research was conducted in the absence of any commercial or financial relationships that could be construed as a potential conflict of interest.
